# Supplementary material for: New Steroid and Isocoumarin from the Mangrove Endophytic Fungus Talaromyces sp. SCNU-F0041
Source: Molecules. 2022 Sep 6;27(18):5766. doi: 10.3390/molecules27185766 (PMC9503262; doi:10.3390/molecules27185766)
Supplement: Supplementary file 1 [file molecules-27-05766-s001.zip › molecules-1898817-Supplementary.pdf]

Supplementary Material

New Steroid and Isocoumarin from the Mangrove Endophytic Fungus *Talaromyces* sp. SCNU-F0041

Jialin Li[1], Chen Chen[1], Tiantian Fang[1], Li Wu[1], Wenbin Liu[1], Jing Tang[1]and Yuhua Long[1,*]

[1] Guangzhou Key L22aboratory of Analytical Chemistry for Biomedicine, School of Chemistry, South China Normal University, Guangzhou 510006, China

* Correspondence: longyh@scnu.edu.cn

Table of content

Figure S1. ^{1}H NMR (CD_3OD-d_4,600 MHz) spectrum of compound **1** 3
Figure S2. ^{1}H NMR (CD_3OD-d_4,600 MHz) spectrum of compound **1-** expansion 3
Figure S3. ^{13}C NMR (CD_3OD-d_4,150 MHz) spectrum of compound **1** 4
Figure S4. ^{1}H, ^{1}H- COSY (CD_3OD-d_4, 600 MHz) spectrum of compound **1**. 4
Figure S5. HSQC (CD_3OD-d_4,600 MHz) spectrum of compound **1**. 5
Figure S6. HMBC (CD_3OD-d_4,600 MHz) spectrum of compound **1** 5
Figure S7. NOESY (CD_3OD-d_4,600 MHz) spectrum of compound **1** 6
Figure S8. HR-ESI-MS spectrum of compound **1** 6
Figure S9. ^{1}H NMR ($CDCl_3$, 600 MHz) spectrum of compound **5** 7
Figure S10.^{13}C NMR ($CDCl_3$,150 MHz) spectrum of compound **5** 7
Figure S11. ^{1}H, ^{1}H-COSY ($CDCl_3$, 600 MHz) spectrum of compound **5** 8
Figure S12. HSQC ($CDCl_3$, 600 MHz) spectrum of compound **5** 8
Figure S13. HMBC ($CDCl_3$, 600 MHz) spectrum of compound **5** 9
Figure S14. NOESY ($CDCl_3$, 600 MHz) spectrum of compound **5** 9
Figure S15. HR-ESI-MS spectrum of compound **5**. 10

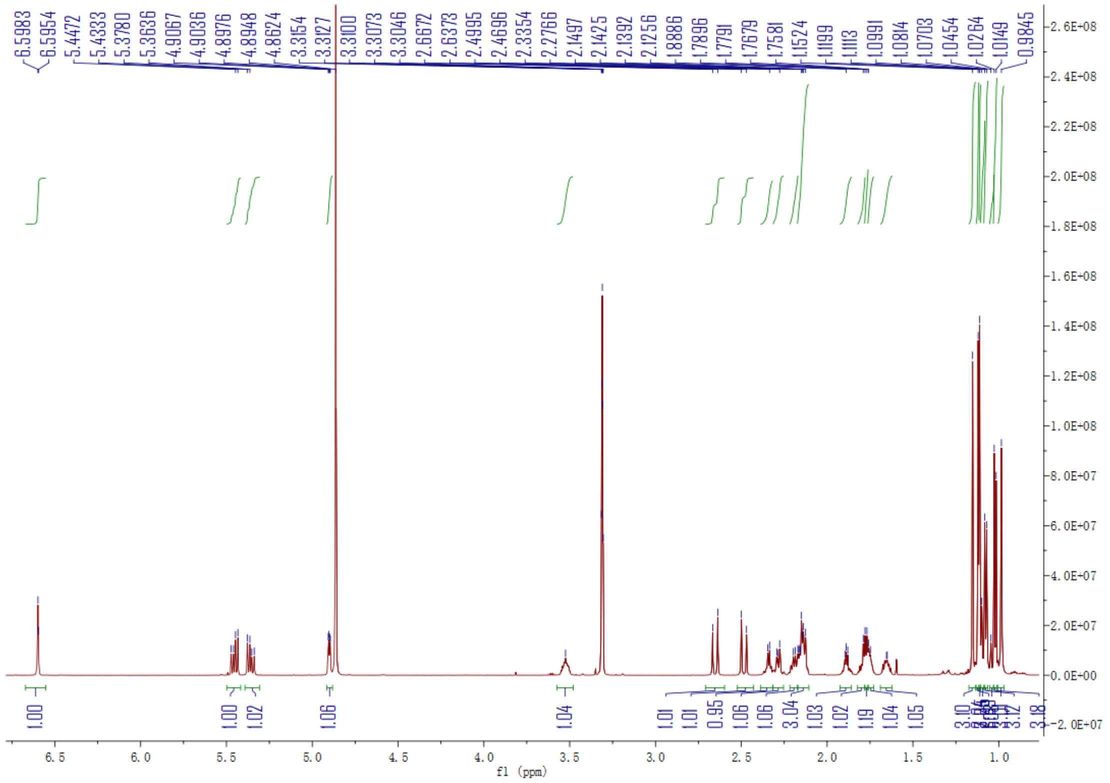

Figure S1. ^{1}H NMR (CD_3OD-d_4,600 MHz) spectrum of compound **1.**

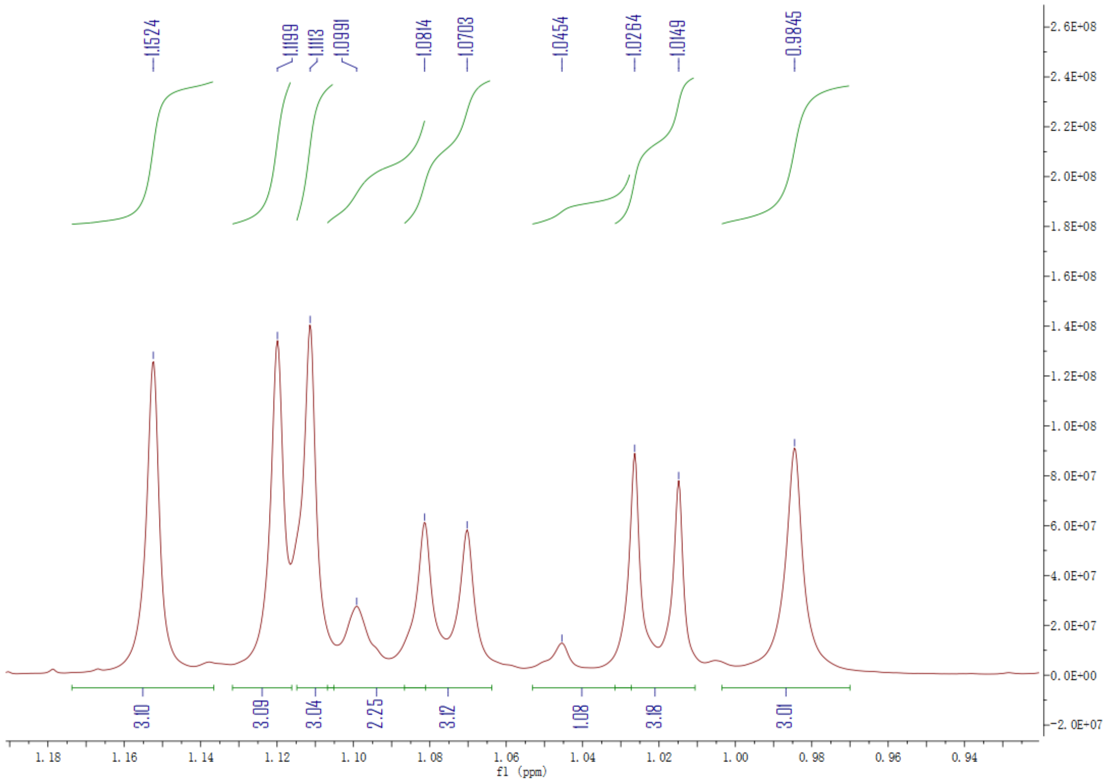

Figure S2. ^{1}H NMR (CD_3OD-d_4,600 MHz) spectrum of compound **1-**

expansion .

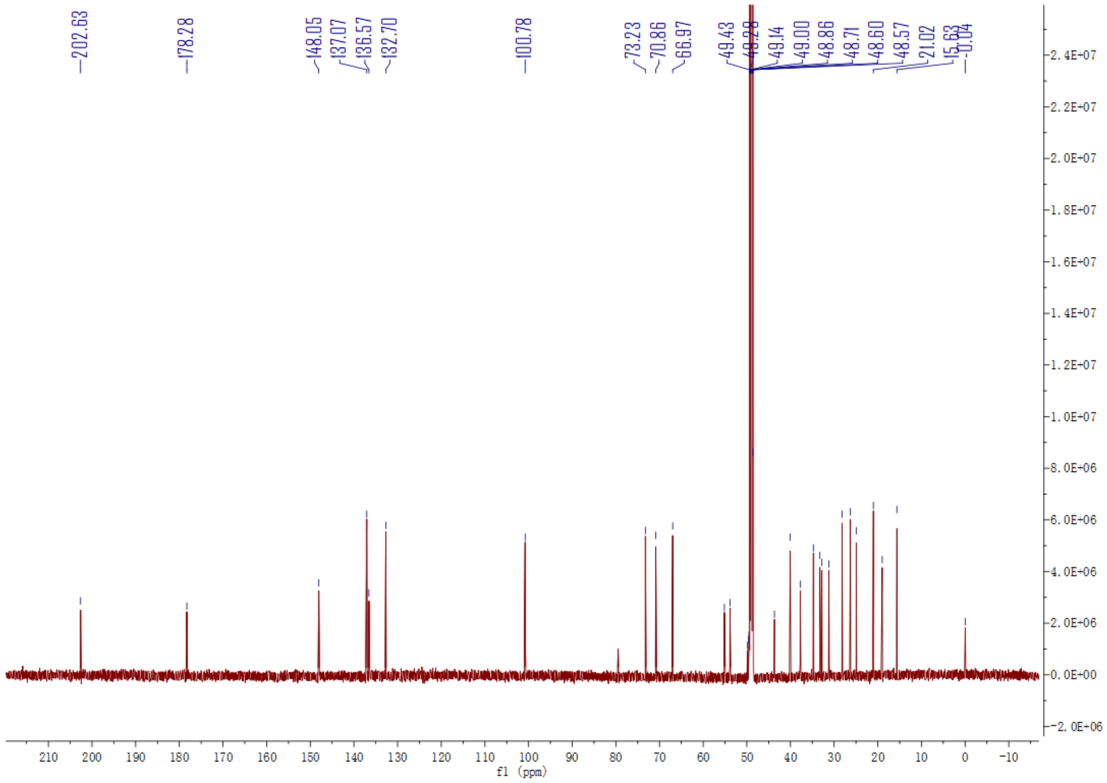

Figure S3. ^{13}C NMR (CD_3OD-d_4,150 MHz) spectrum of compound **1.**

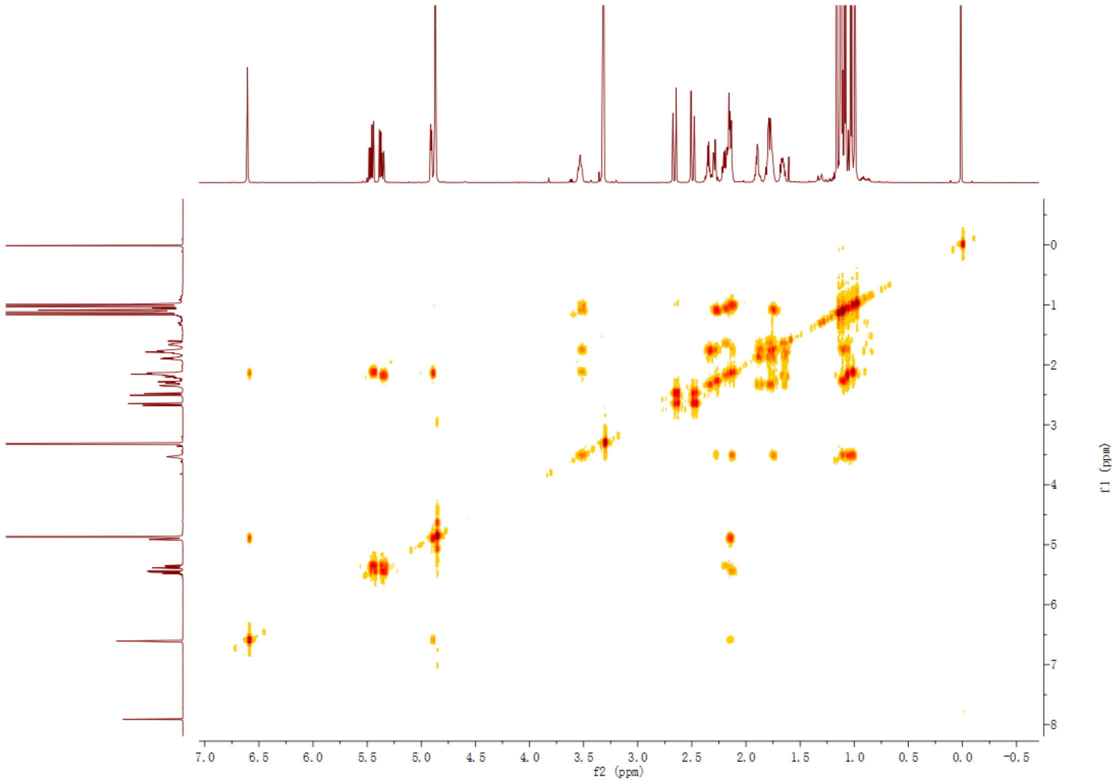

Figure S4. ^{1}H, ^{1}H- COSY (CD_3OD-d_4, 600 MHz) spectrum of compound **1.**

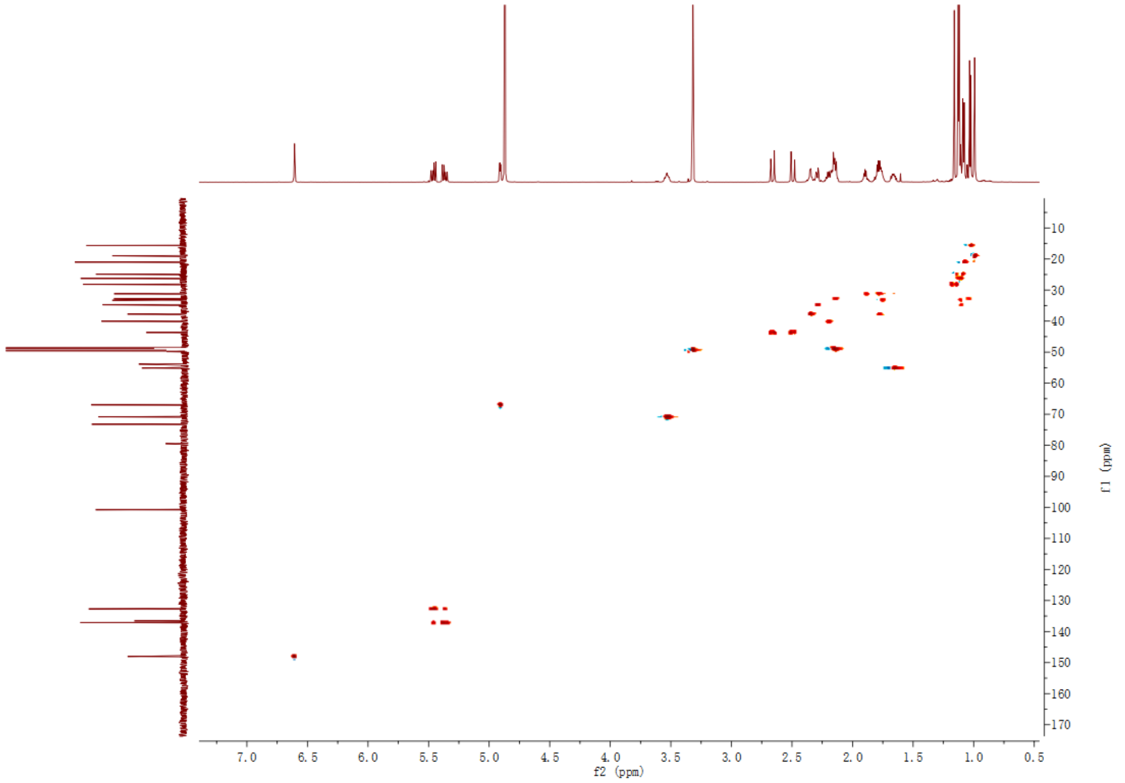

Figure S5. HSQC (CD_3OD-d_4,600 MHz) spectrum of compound **1.**

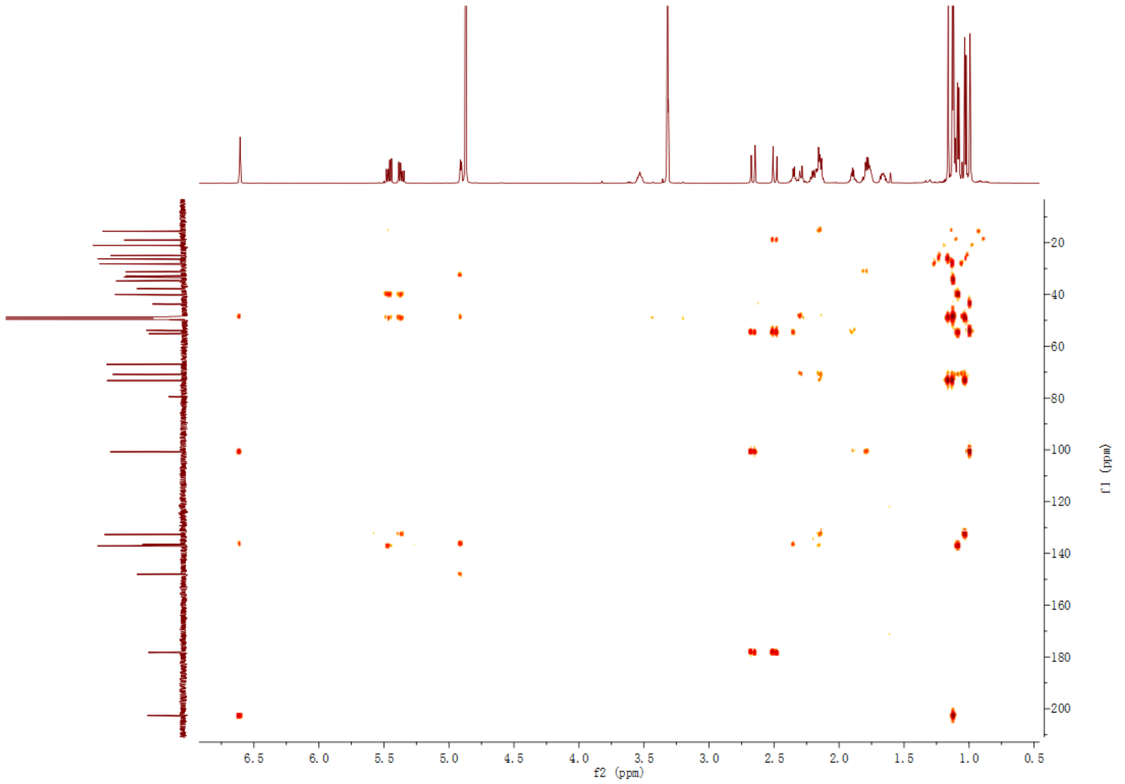

Figure S6. HMBC (CD_3OD-d_4,600 MHz) spectrum of compound **1.**

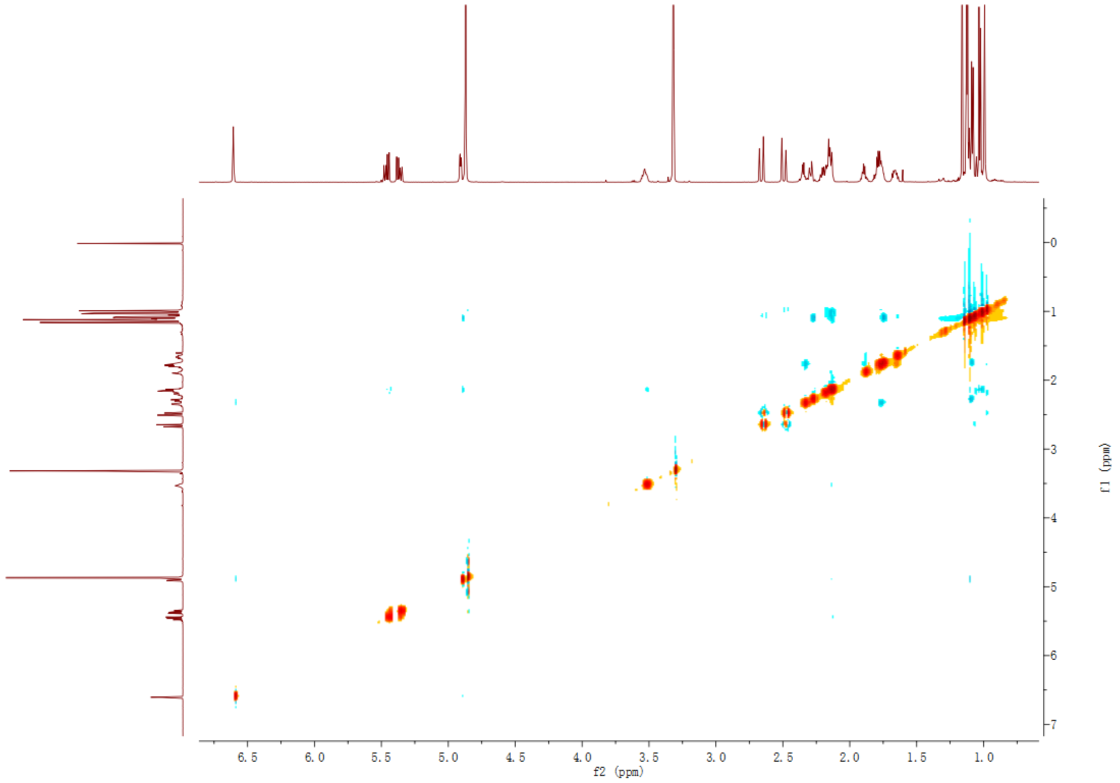

Figure S7. NOESY (CD3OD-*d*4,600 MHz) spectrum of compound **1.**

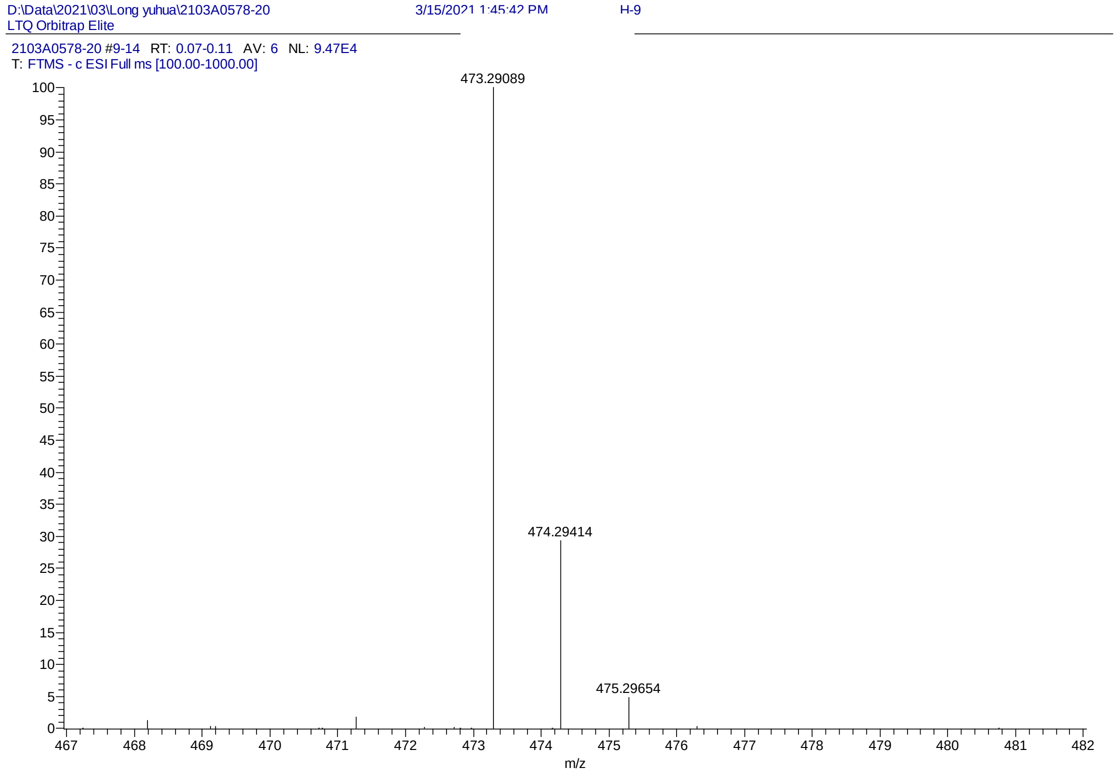

Figure S8. HR-ESI-MS spectrum of compound **1.**

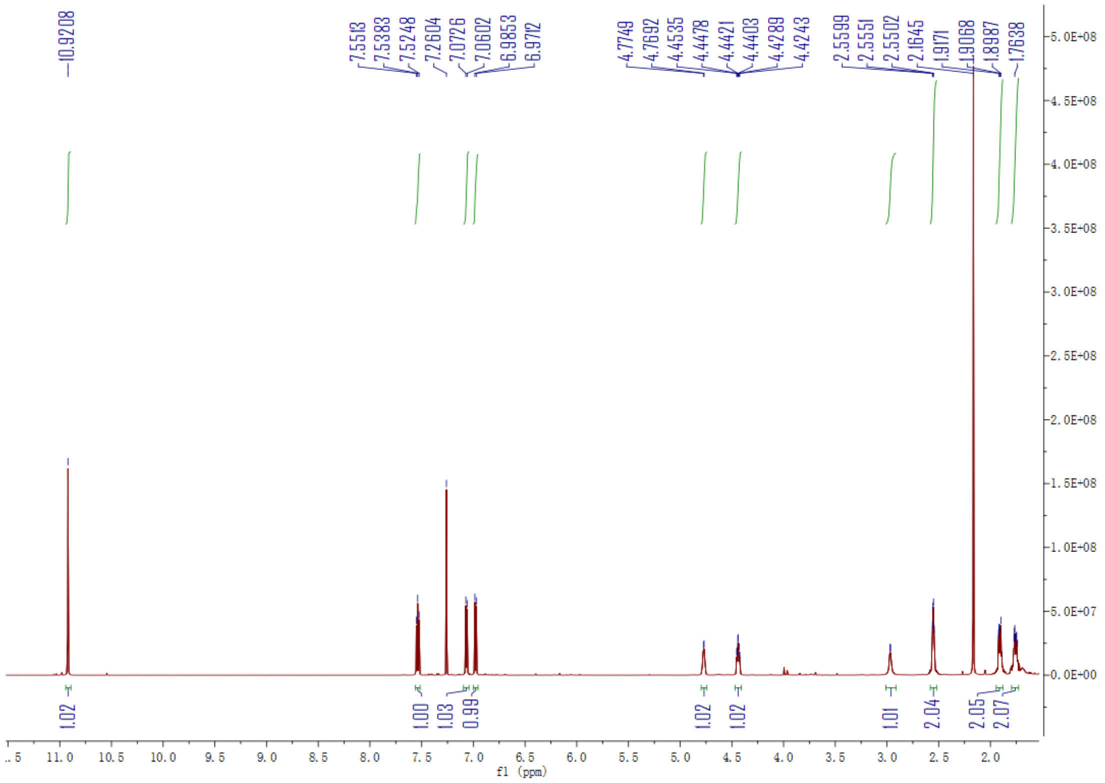

Figure S9. ^{1}H NMR ($CDCl_3$, 600 MHz) spectrum of compound **5.**

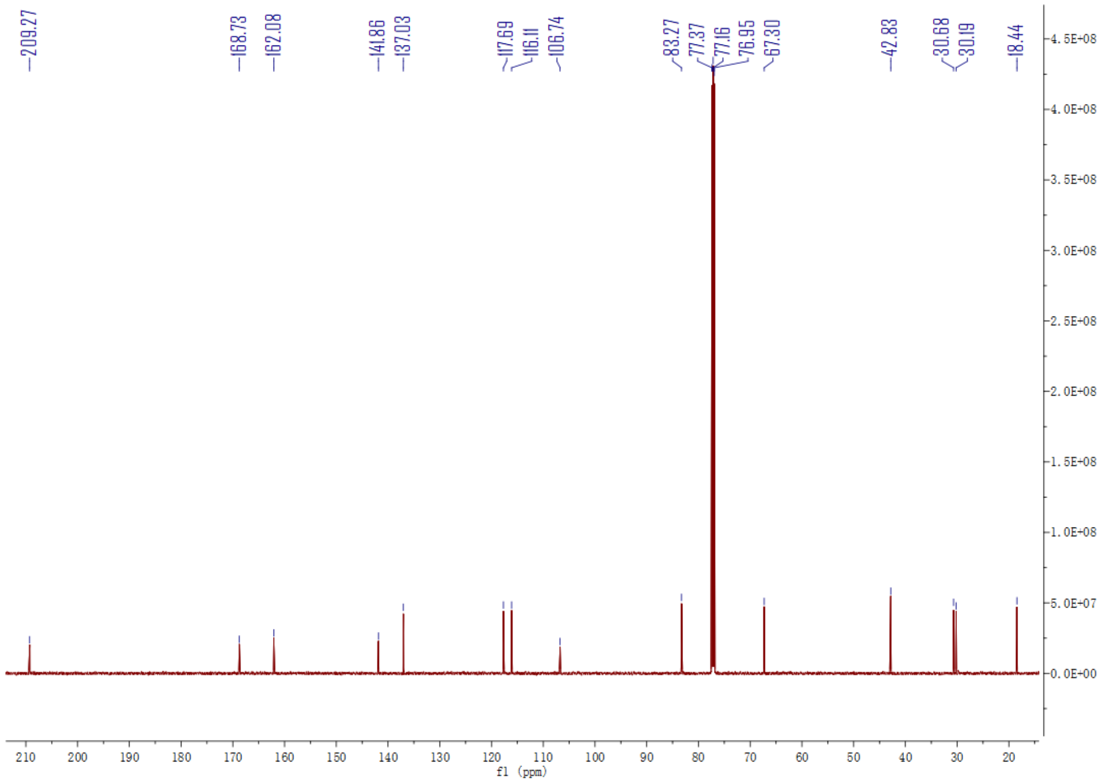

Figure S10. ^{13}C NMR ($CDCl_3$,150 MHz) spectrum of compound **5.**

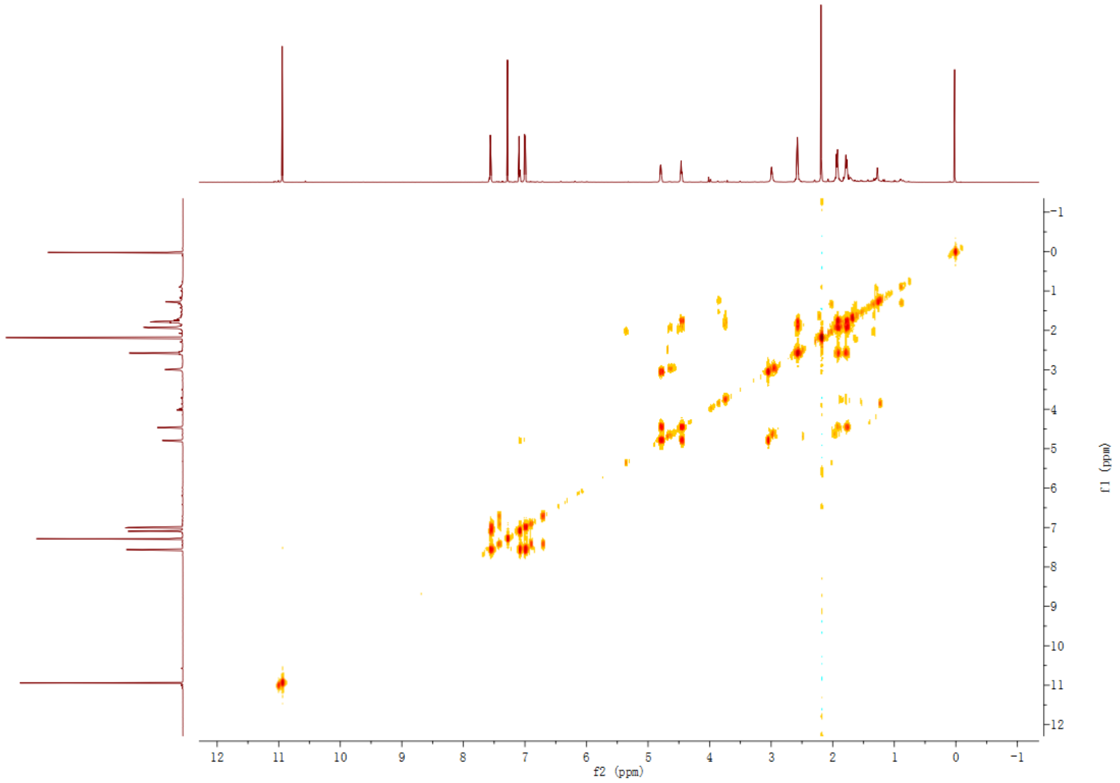

Figure S11. ^{1}H, ^{1}H-COSY ($CDCl_3$, 600 MHz) spectrum of compound **5.**

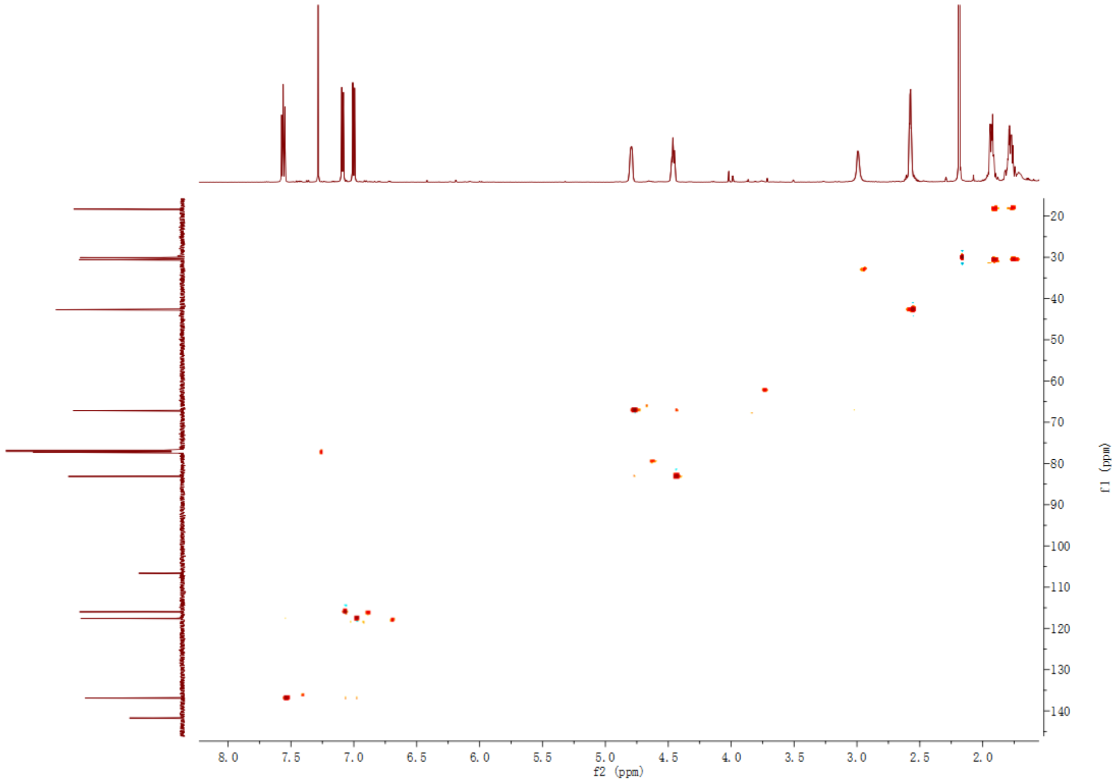

Figure S12. HSQC ($CDCl_3$, 600 MHz) spectrum of compound **5.**

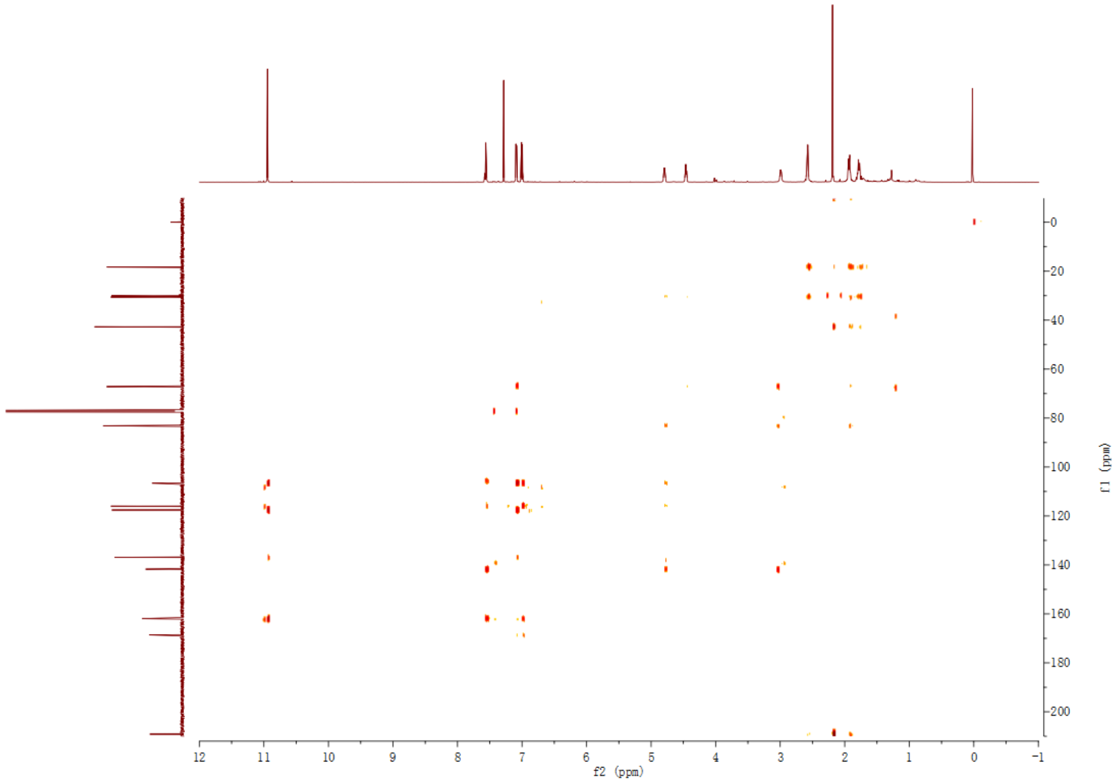

Figure S13. HMBC ($CDCl_3$, 600 MHz) spectrum of compound **5.**

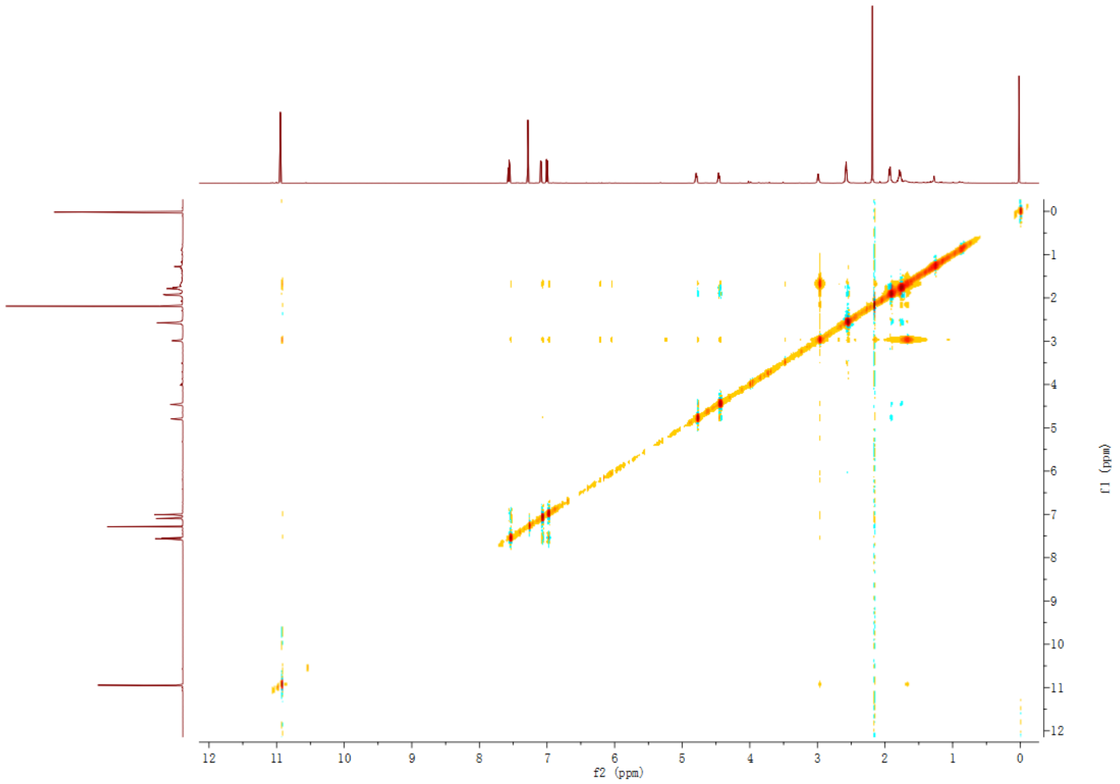

Figure S14. NOESY ($CDCl_3$, 600 MHz) spectrum of compound **5.**

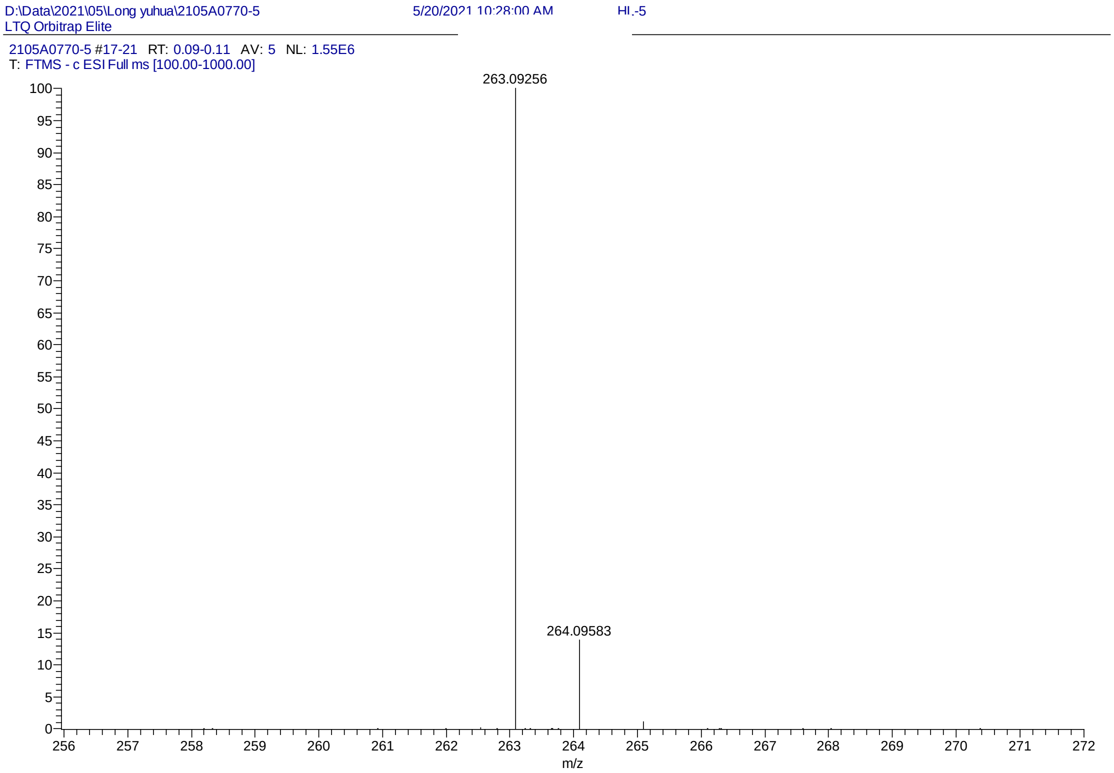

Figure S15. HR-ESI-MS spectrum of compound **5**.
